# The invasive* Aedes albopictus* in the Doñana World Heritage Site

**DOI:** 10.1186/s13071-024-06438-8

**Published:** 2024-08-17

**Authors:** Josué Martínez-de la Puente, Sergio Magallanes, Mikel Alexander González, María José Ruiz-López, Ramón C. Soriguer, Francisco Caceres, Santiago Ruiz, Jordi Figuerola

**Affiliations:** 1 Estación Biológica de Doñana (EBD, CSIC), Seville, Spain; 2 Servicio de Control de Plagas, Diputación Provincial de Huelva, Huelva, Spain; 3 Ciber de Epidemiología y Salud Pública (CIBERESP), Huelva, Spain

**Keywords:** Asian tiger mosquito, First report, Invasive mosquitoes, Mosquito management, Mosquito surveillance, Vector-borne diseases, Wetlands

## Abstract

**Background:**

*Aedes albopictus* is catalogued as one of the 100 most dangerous species worldwide. Native to Asia, the species has drastically increased its distribution range, reaching all continents except Antarctica. The presence of *Ae*. *albopictus* in Spain was first reported in 2004 in Cataluña (NE Spain), and it is spreading in the country.

**Methods:**

We conducted an extensive mosquito monitoring study in the natural protected area of the Doñana National Park (SW Spain) in 2023. After identifying the presence of *Ae*. *albopictus*, a mosquito control strategy was developed and implemented to eradicate the species in the area.

**Results:**

Overall, 12,652 mosquito females of 14 different species were captured at nine sites within the park. For the first time, the presence of *Ae*. *albopictus* was recorded in the area, despite intensive trapping performed at some localities since 2003. The presence of this invasive species in the park is most likely linked to human activities, potentially facilitated by daily car trips of personnel. Although larvae of *Culex*, *Anopheles*, and *Culiseta* mosquitoes were identified in these containers, the presence of *Ae*. *albopictus* larvae was not recorded in those locations. In spite of that, the biological larvicide *Bacillus thuringiensis israelensis* (Bti) was applied to artificial containers potentially used by *Ae*. *albopictus* as breeding sites.

**Conclusions:**

This work evidences the high capacity of *Ae*. *albopictus* to reach highly conserved natural areas far from urban foci. We discuss the implications of the presence of *Ae*. *albopictus* in this endangered ecosystem and the potential control measures necessary to prevent its reintroduction.

**Graphical Abstract:**

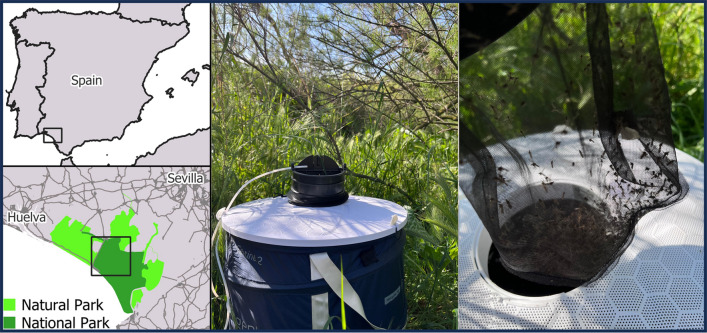

## Background

Mosquitoes and the pathogens they transmit are significant global health concerns, causing fatalities and economic impacts worldwide [1]. Favored by human activities, different mosquito species have been introduced and established in new areas, affecting the local transmission of vector-borne pathogens. This is the case of invasive mosquitoes of the *Aedes* genus. Their expansion has changed the epidemiological scenarios of various pathogens in the new areas they have invaded. Additionally, invasive *Aedes* mosquitoes, owing to their cumulative damage and management costs, represent the highest economic impact among known invasive taxa [2].

The Asian tiger mosquito, *Aedes albopictus,* is a widespread mosquito species catalogued as one of the 100 worst invasive species [3]. Native to Southeast Asia, *Ae*. *albopictus* has significantly expanded its distribution in recent decades. International trade of used tires and lucky bamboo, among other goods, has contributed to the global spread of *Ae*. *albopictus* [4]. In Europe, this species was first detected in Albania in 1979 and nowadays is established in many countries in the continent, especially in the Mediterranean basin. In 2004, *Ae*. *albopictus* was first identified in Spain, in an area of Cataluña (NE Spain), and since then it has progressively colonized several areas, including Southern Spain (e.g., Andalusia) [5, 6]. *Aedes albopictus* represents a major threat to public health as it is a competent vector of viruses causing dengue, Zika, and Chikungunya [7, 8], as well as zoonotic parasites, such as *Dirofilaria* [9], enabling local transmission of these pathogens in the invaded areas.

In 2023, we conducted a mosquito monitoring study across different areas of the protected wetland of the Doñana National and Natural Park tracking mosquito populations in the region. Upon detecting the invasive *Ae*. *albopictus* during this surveillance, we implemented control measures, in coordination with the authorities of this protected area.

## Methods

### Study area, mosquito trapping, and identification

**Fig. 1:**
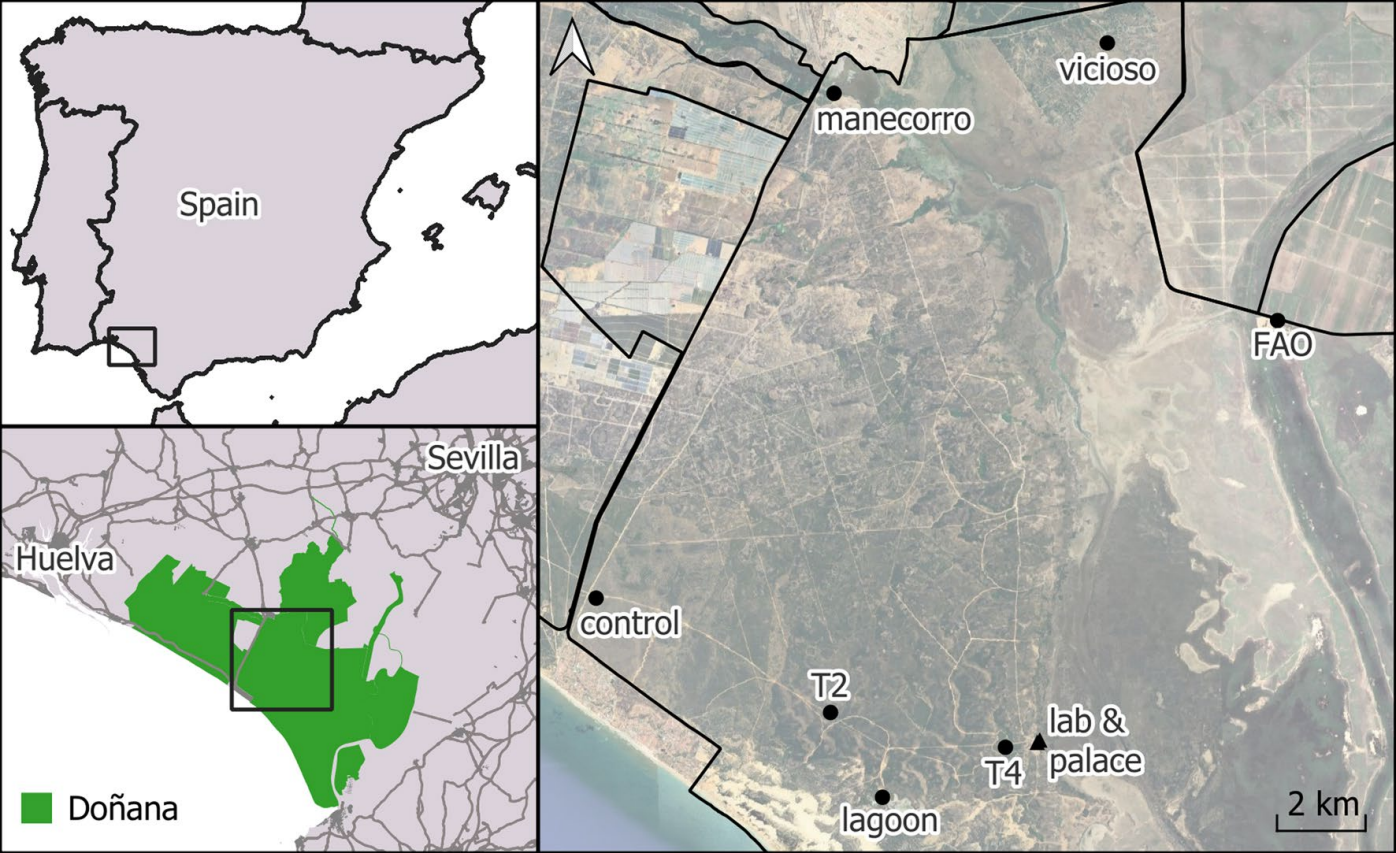
Sampling sites in the Doñana National and Natural Park. Sampling localities negative (*n* = 7) and positive (*n* = 2) for the presence of *Aedes albopictus* are marked with circles and triangles, respectively. Note that the two positive localities were so close that the symbols overlap

This study was conducted in the World Heritage Site of Doñana National and Natural Park, Southern Spain (Fig. 1). This exceptional wetland is characterized by a typical Mediterranean climate, featuring a long dry summer season with most precipitation occurring during winter and spring. Mosquito trapping was conducted using two Biogents BG-sentinel-2 traps at each of the nine sampling sites within the park. Traps were baited with ca. 1 kg of dry ice as a source of CO_2_ for mosquito attraction. At each sampling site, mosquito traps operated for 24 h. Sampling sites were selected on the basis of accessibility and to represent areas with different environmental characteristics within the park. In addition, we included sampling sites that were sampled in previous studies conducted in the area. Four trapping sessions were conducted in this study, one session every 45 days, from April to September 2023. Adult mosquitoes were preserved in dry ice in the field and stored frozen at −80ºC until morphological identification in the laboratory. Subsequently, mosquitoes were sorted by sex and date of collection on a chill table, and then identified to the species level on the basis of their morphology ([10]; see further details of the procedure in [11]). After identifying *Ae*. *albopictus* in this area (see Results), a control plan was developed. Technicians from the *Servicio de Control de Plagas* of the *Diputación de Huelva* identified mosquito larvae present in the artificial water containers at the positive sites. They applied Bti (VECTOBAC 12 AS) at a concentration of 20 ml/l to artificial water containers present in these positive sites.

## Results

In total, 12,652 mosquito females belonging to 14 different species and four genera were captured (Table 1). During this study, three traps failed to work during a single trapping session, and the very few mosquitoes retained in these traps were not taken into consideration. The most common mosquito species was *Aedes* (*Ochlerotatus*) *caspius* (*n* = 8794 females), representing 69.5% of the mosquito females captured. The number of mosquito females captured for the remaining species are shown in Table 1. In total, 121 males were captured in the present monitoring, including 55 *Culex theileri*, 31 *Anopheles atroparvus*, 18 *Cx*. *pipiens*, 9 *Ae*. *caspius*, 5 *Culiseta longiareolata*, 1 *Cx*. *modestus*, and 1 *Ae*. *vexans*. In addition, 1 male and 13 females of *Ae*. *albopictus* were trapped at two sampling sites of the Doñana National Park, namely Palacio de Doñana and the Lucio del Palacio (Fig. 1). These sites were relatively close to each other (300 m, approximately). All *Ae*. *albopictus* mosquitoes were captured during the trapping sessions conducted in August and September.

**Table 1:** Number of mosquito females captured in this study

Species	Lucio del Palacio	RBD Control	RBD2	RBD4	Arroyo de la Cañada Mayor	C.V. José A. Valverde	Santa Olalla	Manecorro	Palacio de Doñana	Total
*Aedes* (*Aedimorphus*) *vexans*	9									9
*Aedes* (*Ochlerotatus*) *berlandi*								1		1
*Aedes* (*Ochlerotatus*) *caspius*	148	391	364	1358	81	43	5903	208	298	8794
*Aedes* (*Ochlerotatus*) *detritus*	43	4	19	188	1	70	218		151	694
*Aedes (Stegomyia) *albopictus	12								1	13
*Aedes* sp.				1						1
*Anopheles algeriensis*						32				32
*Anopheles atroparvus*	31	7	6	33	69	70	9	10	31	266
*Culex laticintus*		3	1							4
*Culex modestus*	2			2	1	133	7	2		147
*Culex perexiguus*	4	2		3	745	62	25	38	3	882
*Culex pipiens*	71	126		2	26	185	10	85	28	533
*Culex theileri*	36	9	4	11	26	583	251	247	88	1255
*Culiseta annulata*				1		3		5		9
*Culiseta longiareolata*	1	7							4	12

**Fig. 2:**
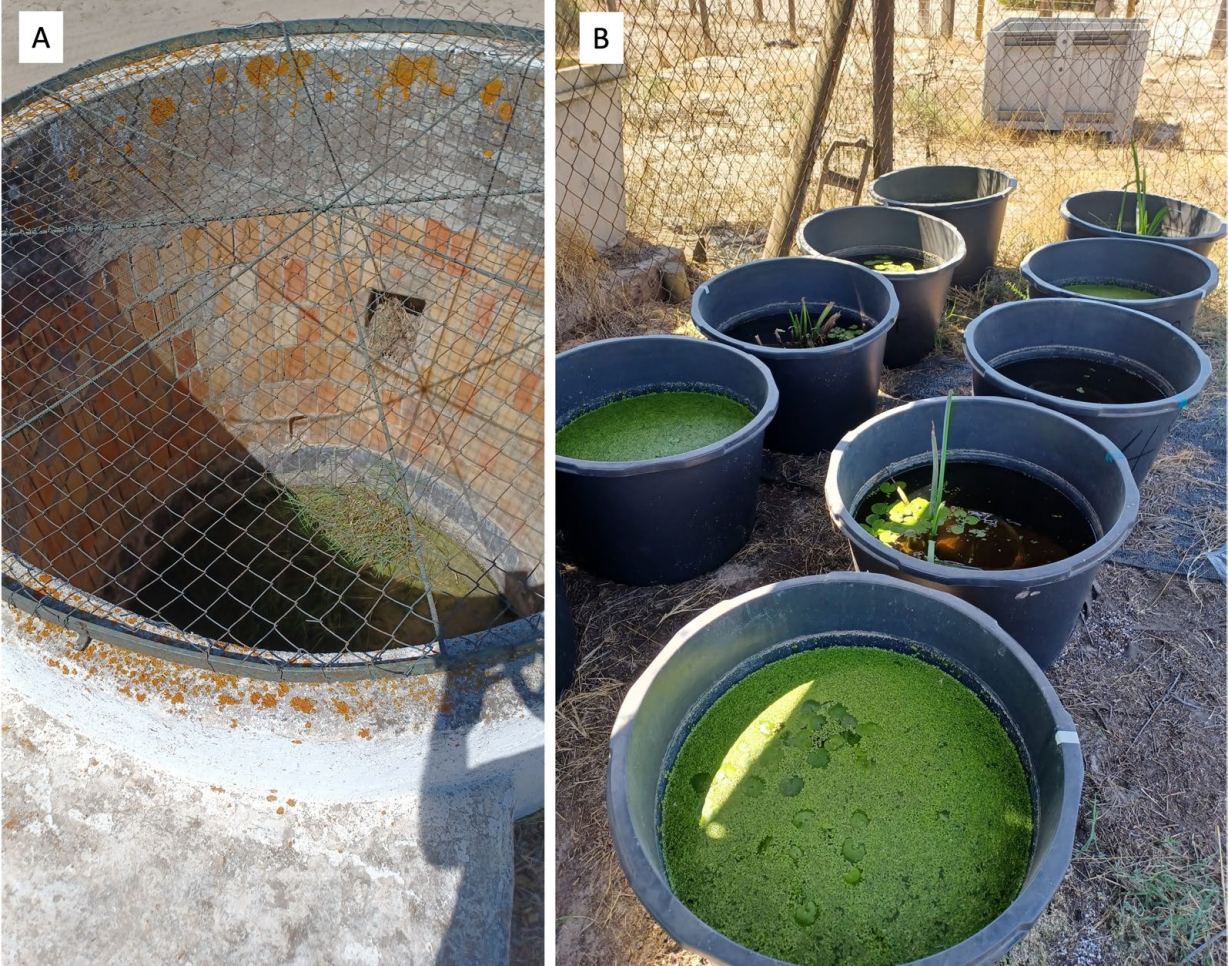
Water well **A** and artificial water-holding containers **B** around the positive traps used by mosquitoes as breeding sites in the Doñana National Park

Bti treatments were applied in two sessions, on 21 August and 18 September, to artificial water containers at the two positive sites (Fig. 2). Entomological inspections of water containers behind Lucio del Palacio identified *Culex perexiguus* larvae, while larvae of the species *Cx*. *pipiens* s.l., *Cx*. *theileri*, *Culex laticinctus*, *An*. *atroparvus*, and *Cs*. *longiareolata* were found in Palacio de Doñana. The presence of *Ae*. *albopictus* larvae was not recorded in these places.

## Discussion

The continuous monitoring of mosquitoes in the Doñana National and Natural Park in 2023 allowed the identification of 14 different mosquito species belonging to the genera *Aedes*, *Anopheles*, *Culex*, and *Culiseta*. In this area, mosquito monitoring has been routinely conducted since 2003 [12]; however, the presence of the invasive species *Ae*. *albopictus* in this highly protected natural area has not been reported until now.

The passive transport of *Ae*. *albopictus* by cars has been demonstrated in Spain [13], which may also explain its introduction in Doñana. Adult mosquitoes could be transported in vehicles from surrounding areas where this species is already established. According to the citizen science platform Mosquito Alert (https://www.mosquitoalert.com), there was a significant increase in *Ae*. *albopictus* reports rising from 19 in 2022 to 200 in 2023 in the surrounding provinces of Sevilla and Huelva. Another possible explanation is the local transport of material (i.e., containers, pots, or other goods) harboring the eggs or immature stages of *Ae*. *albopictus*. Eggs of *Ae*. *albopictus* are highly resistant to desiccation and shipping, allowing the viability of specimens transported from one site to another. Although we did not detect *Ae*. *albopictus* larvae, suitable habitats for the development of the larvae of this species mostly include artificial water containers, although *Ae*. *albopictus* can also use natural breeding sites [14]. *Aedes albopictus* females feed on blood from a wide range of vertebrates, although mammals, including humans, dominate their diet [15]. Humans are present in the areas where *Ae*. *albopictus* mosquitoes were found in Doñana, especially during the day when the species reaches its maximum activity. The potential role of this mosquito species in the local transmission of pathogens in the studied area, including zoonotic ones, merits further investigation. This includes *Dirofilaria immitis*, which may circulate in Doñana and affect highly endangered species, such as the Iberian lynx *Lynx pardinus* [16]. West Nile and Usutu viruses also circulate in the area [17] and could be transmitted by *Ae*. *albopictus* [14], although the role of this species in the transmission of avian pathogens is likely low owing to its highly mammal-biased blood-feeding pattern [15, 18].

Because *Ae*. *albopictus* colonization of the area monitored in this study should be at an initial stage, local authorities authorized an urgent control program to prevent or minimize spread. In our study, we applied Bti in water-holding containers at two time points to inhibit the development of mosquito larvae in artificial containers used as potential larval sites. Although in this study we collected mosquitoes until September, additional captures done in the Palacio de Doñana station using Center for Disease Control (CDC) traps supplemented with CO_2_ in September and October did not detect the presence of *Ae*. *albopictus*. While these actions may have contributed to limiting the establishment of *Ae*. *albopictus* in the area where *Ae*. *albopictus* was found, future reintroductions should be considered. Therefore, it is necessary to define future monitoring recommendations and control measures to reduce the chances of establishment. For example, it is important to clean and sterilize any container introduced in the area that can retain water using diluted bleach, and to cover all the tanks used for microcosm experiments with water with mosquito screens. However, the main routes of *Ae*. *albopictus* introduction into the Doñana National and Natural Park are probably the vehicles used by researchers, workers, and park visitors, making extremely difficult to reduce this source of introduction. A possible solution for minimizing the impact of this kind of importation would be the spraying of commercial insecticides inside vehicles before displacements, especially during the months of peak activity of *Ae*. *albopictus*. This may help minimizing this form of introduction.

## Data Availability

Data analyzed in this study are included in this published article.
